# Investigation of coenzyme Q10 status, serum amyloid-β, and tau protein in patients with dementia

**DOI:** 10.3389/fnagi.2022.910289

**Published:** 2022-07-25

**Authors:** Po-Sheng Chang, Hsi-Hsien Chou, Te-Jen Lai, Chi-Hua Yen, Ji-Cyun Pan, Ping-Ting Lin

**Affiliations:** ^1^Department of Nutrition, Chung Shan Medical University, Taichung, Taiwan; ^2^Graduate Program in Nutrition, Chung Shan Medical University, Taichung, Taiwan; ^3^School of Medicine, Chung Shan Medical University, Taichung, Taiwan; ^4^Department of Neurology, Chung Shan Medical University Hospital, Taichung, Taiwan; ^5^Institute of Medicine, Chung Shan Medical University, Taichung, Taiwan; ^6^Department of Psychiatry, Chung Shan Medical University Hospital, Taichung, Taiwan; ^7^Department of Family and Community Medicine, Chung Shan Medical University Hospital, Taichung, Taiwan; ^8^Department of Nutrition, Chung Shan Medical University Hospital, Taichung, Taiwan

**Keywords:** coenzyme Q10, antioxidant capacity, amyloid-β, tau protein, dementia

## Abstract

**Objectives:**

Dementia is an oxidative stress-related disease. Coenzyme Q10 is a nutrient that occurs naturally in the human body and acts as an antioxidant. The purpose of this study was to investigate the relationships of coenzyme Q10 status, biomarkers for dementia (amyloid β and tau protein), and antioxidant capacity in patients with dementia.

**Methods:**

Eighty dementia patients aged ≥60 years and with a mini mental state examination (MMSE) score ≤ 26 were enrolled. The levels of coenzyme Q10, total antioxidant capacity (TAC), amyloid β, and tau protein were measured.

**Results:**

A total of 73% of patients had a low coenzyme Q10 status. Patients with low coenzyme Q10 status had a significantly higher level of serum amyloid β-42 and amyloid β-42/40 ratio (*p* < 0.05). Coenzyme Q10 status was significantly correlated with the values of TAC, MMSE score, amyloid β-42, and amyloid β-42/40 ratio (*p* < 0.05) but not with tau protein. Additionally, a high proportion of moderate dementia patients were found to have low coenzyme Q10 status (*p* = 0.07).

**Conclusion:**

Patients with dementia suffered from coenzyme Q10 deficiency, and the degree of deficiency was related to the level of amyloid-β and antioxidant capacity. Since adequate level of coenzyme Q10 may delay the progression of dementia, monitoring coenzyme Q10 status in patients with dementia is necessary.

## Introduction

Dementia has become a critical public health issue. The latest report of the World Health Organization estimated that more than 55 million people are living with dementia worldwide, and the number is growing every year (WHO, 2021). The pathophysiology of dementia is credited to the extracellular growth of amyloid-β oligomers causing insoluble plaques and the hyperphosphorylation of tau protein that produces neurofibrillary tangles within neuron cells, which damages synapses that mediate memory and cognition (Bloom, 2014). Dementia is also an oxidative stress-related and mitochondrial dysfunction disease (Cabezas-Opazo et al., 2015; Tönnies and Trushina, 2017). Patients with dementia have a higher level of oxidative stress and reduced antioxidant defenses, which changes the synaptic activity and neurotransmitter release (Tönnies and Trushina, 2017). Thus, researchers have suggested that antioxidants play the role in delaying the progression of dementia (Sinyor et al., 2020). Antioxidant nutrients, such as vitamins C and E have been shown to reduce amyloid-β-induced oxidative stress (Basambombo et al., 2017; Gugliandolo et al., 2017). Lipophilic antioxidants, such as vitamin A, vitamin E, and carotenoids, play the role of preventing cell membranes from damage by free radicals and may aid in halting the progression of neurodegeneration (Chang et al., 2018). A review of human studies indicated that lipid peroxidation involved with mitochondrial dysfunction was a factor in the pathogenesis of neurodegenerative diseases, which may be decreased by the lipophilic antioxidants to reduce the severity of neurodegenerative diseases (Petrovic et al., 2020). Coenzyme Q10 is also a lipophilic antioxidant in the mitochondria, which may react with reactive oxygen species directly. In addition to traditional antioxidants (vitamins), the effect of coenzyme Q10 on neurodegenerative disease has begun to be discussed (Spindler et al., 2009; Yang et al., 2016).

Coenzyme Q10 is an essential electron carrier in the mitochondrial electron transport chain for the generation of adenosine triphosphate (Bentinger et al., 2010). Coenzyme Q10 can act as an antioxidant against the increase in reactive oxygen species during the development of chronic diseases (Tönnies and Trushina, 2017). Studies have shown that patients with chronic diseases including, neurodegenerative diseases, cardiovascular disease, and cancer, have lower coenzyme Q10 status (Arenas-Jal et al., 2020). Yamagishi et al. (2014) were the first to observe that the level of coenzyme Q10 was associated with the risk of dementia. The neuroprotective effect of coenzyme Q10 has been demonstrated in animal models (Komaki et al., 2019; Ibrahim Fouad, 2020). However, few human studies have examined the correlation between the level of coenzyme Q10 and biomarkers for dementia (such as amyloid β and tau protein) in clinical setting. Therefore, the purpose of this study was to investigate the relationships of coenzyme Q10 status, amyloid β and tau protein, and antioxidant capacity in patients with dementia.

## Materials and methods

### Participants and study design

Eighty patients with dementia were recruited from the Chung Shan Medical University Hospital, which is a medical center in Taiwan. Patients with dementia were diagnosed by a neurologist and psychiatrist based on brain magnetic resonance imaging or computed tomography scan to confirm the absence of structural lesions. The inclusion criteria were age ≥60 years and mini mental state examination (MMSE) score ≤ 26. The MMSE questionnaire included six domains, such as orientation, registration, attention and calculation, recall, language, and visual construction. The sum of all item scores on the MMSE was 30, with higher scores indicating better cognitive function. As an MMSE score > 20 was defined as mild dementia and an MMSE score ≤ 20 was defined as moderate dementia (Folstein et al., 1975; Nolan et al., 2018). We excluded patients who were diagnosed with cancer, severe heart, lung, liver, and kidney disease, severe disability or aphasia, and the use of coenzyme Q10 supplements. A total of eighty patients with dementia met the criteria of the study, and all of them completed the measurement and examination. This study was approved by the Institutional Review Board of Chung Shan Medical University Hospital, Taiwan (CSMUH No: CS2-18147). Informed consent was obtained from each subject before participating in the present study.

### Demographic data collection

The demographic data of patients with dementia, including age and gender, were collected by a questionnaire. A digital electronic sphygmomanometer (Hartmann Tensoval^®^ duo control, Heidenheim, Germany) was used to measure blood pressure. Height and weight were measured to calculate body mass index (BMI). Waist circumference was measured with a tape measure. In addition, we used vacutainers with K2-EDTA anticoagulant (Becton Dickinson, Franklin Lakes, NJ, United States) or without anticoagulant to collect the venous blood sample after 12 h of fasting in patients with dementia. Plasma, serum, and red blood cell (RBC) samples were separated after centrifugation at 3,000 rpm for 15 min at 4°C. The white blood cell (WBC) sample was collected by using RBC lysis buffer and stored at −80°C until analysis. An automated chemistry analyzer (Roche, Cobas c501, Risch-Rotkreuz, Switzerland) was used to analyze the levels of triglycerides (TG), total cholesterol (TC), low density lipoprotein-cholesterol, high density lipoprotein-cholesterol (HDL-C), fasting glucose, glycated hemoglobin, and high-sensitivity C-reactive protein.

### Measurement of coenzyme Q10 status

High performance liquid chromatography (HPLC) with an ultraviolet detector was used to measure the level of coenzyme Q10 in plasma and WBC (Littarru et al., 2007). The WBC pellet was homogenized by adding 1-propanol in preparation for coenzyme Q10 measurement. The coenzyme Q10 in plasma and homogeneous fluid of WBC was extracted by 1-propanol. After centrifugation at 12,000 rpm for 15 min, the supernatant was mixed with methanol for a ratio of 1:1 and then filtered with a PTFE syringe filter, 0.45 μm × 13 mm (Branch Billion Lung, Tianjin, China) for analysis. Mixed methanol and ethanol were used as the mobile phase, and the flow rate was set at 0.8 mL/min. The analysis column was a LiChroCART^®^RP-18 (Merck, Darmstadt, Germany), and the wavelength of the detector was set at 275 nm. The standard of coenzyme Q10 was purchased from Sigma-Aldrich (Merck, Germany) as the external standard to apply the calibration curve to measure the level of coenzyme Q10. In the method of coenzyme Q10 in this study, the linearity with correlation coefficients of the standard curve was 0.9999. The mean of intra- and inter-assay coefficients of variability for coenzyme Q10 were 3.4 and 2.8%, respectively. The mean analytical recovery of coenzyme Q10 was 102.0%. The reference range of plasma coenzyme Q10 was 0.5–1.7 μM in adults, and the individuals with plasma coenzyme Q10 level lower than 0.50 μM were defined as having plasma coenzyme Q10 deficiency (Molyneux et al., 2008).

### Total antioxidant capacity measurement

A Trolox equivalent antioxidant capacity assay was used to analyze the total antioxidant capacity (TAC) in serum and RBC (Re et al., 1999). The 2,2’-azinobis (3-ethylbenzothiazoline-6-sulfonic acid) radical was prepared, which exhibited a blue–green color. The level of TAC was measured through the neutralized reaction between antioxidants in serum or free radicals in RBC. A vitamin E analog, Trolox, was used as a standard in the calculation of the level of TAC, which was expressed as mM Trolox. A spectrophotometer was used to measure the absorbance at 730 nm.

### Biomarkers for dementia measurements

We used an enzyme-linked immunosorbent assay kit to quantify the levels of amyloid β-42 (KHB3441, Thermo Fisher Scientific, MA, United States), amyloid β-40 (KHB3481, Thermo Fisher Scientific, MA, United States), and tau protein (KHB0041, Thermo Fisher Scientific, MA, United States) in serum, and followed the manufacturer’s instructions.

### Statistical analysis

All data were analyzed by using SigmaPlot software (version 12.0, Systat, San Jose, CA, United States) in the present study. The mean ± standard deviation (median) or percentages are shown for continuous variables or categorical variables, respectively. The Shapiro−Wilk test was used to examine the normality of distribution for the data. The differences in demographic data, antioxidant capacity, and biomarkers for dementia between the two groups stratified by coenzyme Q10 status were examined by Student’s *t*-test or the Mann−Whitney rank sum test. Spearman’s rank order correlation coefficient was calculated to examine the correlations between coenzyme Q10 status and antioxidant capacity and biomarkers for dementia in patients with dementia. The differences in coenzyme Q10 status between mild and moderate dementia patients were examined by the Mann−Whitney rank sum test; the differences in the proportion of moderate dementia between low and high coenzyme Q10 status were examined by the Chi-square test and the Fisher’s exact test. Statistical significance was set at *p* ≤ 0.05.

## Results

### Demographic data and coenzyme Q10 status in patients with dementia

Table 1 shows the demographic data and coenzyme Q10 status in patients with dementia. In these patients, the median age was 77.0 years, the male to female ratio was 1:3, and the median BMI was 23.8 kg/m^2^. The median values of waist circumference were 91.8 and 89.3 cm in male and female, respectively, which were higher than the reference values for waist circumference (male: < 90 cm, female: < 80 cm). In hematology, the fasting glucose level was higher than the normal reference value (fasting glucose < 5.6 mmol/L). Regarding coenzyme Q10 status, the median level of plasma coenzyme Q10 was 0.36 μM. Approximately 73% of patients with dementia had plasma coenzyme Q10 deficiency, with coenzyme Q10 level lower than 0.50 μM. We therefore stratified by coenzyme Q10 status based on plasma coenzyme Q10 (0.5 μM) and found that patients with low plasma coenzyme Q10 status had significantly lower lipid profiles, such as TC (*p* = 0.02) and HDL-C (*p* < 0.01).

**TABLE 1:** Demographic data and coenzyme Q10 status in patients with dementia.

			Patients with dementia (*N* = 80)
Age (years)			76.5 ± 6.3 (77.0)
Gender (male/female)			22/58
**Physical features**	
SBP (mmHg)			129.6 ± 17.4 (130.0)
DBP (mmHg)			73.0 ± 10.3 (72.5)
BMI (kg/m^2^)			24.2 ± 3.9 (23.8)
Waist circumference (cm)			90.7 ± 10.0 (90.4)
Male			91.3 ± 7.1 (91.8)
Female			90.4 ± 10.9 (89.3)
**Clinical characteristics**	
Triglycerides (mmol/L)			1.4 ± 1.0 (1.3)
Total cholesterol (mmol/L)			4.8 ± 1.0 (4.6)
LDL-C (mmol/L)			3.0 ± 1.0 (2.8)
HDL-C (mmol/L)			1.6 ± 0.5 (1.6)
Male			1.4 ± 0.4 (1.3)
Female			1.7 ± 0.5 (1.6)
Fasting glucose (mmol/L)			6.6 ± 1.6 (6.1)
Glycated hemoglobin (%)			6.3 ± 1.0 (6.0)
Hs-CRP (mg/L)			2.1 ± 5.2 (0.8)
GOT (U/L)			25.7 ± 12.4 (23.0)
GPT (U/L)			19.7 ± 10.8 (17.5)
WBCs (× 10^9^/L)			6.1 ± 1.5 (6.0)
RBCs (× 10^12^/L)			4.5 ± 0.6 (4.5)
Hemoglobin (mmol/L)			13.5 ± 1.4 (13.5)
Hematocrit (%)			39.9 ± 3.8 (40.1)
Platelet (× 10^9^/L)			221.0 ± 64.9 (218.0)
**Comorbidities (n, %)**	
Hypertension			47 (58.8%)
Diabetes			29 (36.3%)
Hyperlipidemia			29 (36.3%)
Heart disease			16 (20.0%)
Liver disease			6 (7.5%)
Chronic kidney disease			5 (6.3%)
**Education (n, %)**	
None			15 (18.8%)
Elementary school			33 (41.3%)
Junior high school			15 (18.8%)
Senior high school			14 (17.5%)
University or above			3 (3.8%)
**Place of residence (n, %)**	
Center			78 (97.5%)
Southern			2 (2.5%)
Use of supplement (n, %)			42 (52.5%)
**Use of hypolipidemic agents (n, %)**	
HMG-CoA reductase inhibitors			30 (37.5%)
Fibric acid derivatives			4 (5.0%)
Cholesterol absorption inhibitor			2 (2.5%)
Nicotinic acid			1 (1.3%)
**Coenzyme Q10 status**	
Plasma coenzyme Q10 (μM)			0.41 ± 0.21 (0.36)
Plasma coenzyme Q10/TC (μmol/mmoL)			0.09 ± 0.04 (0.08)
WBC coenzyme Q10 (nmol/g)			3.4 ± 1.5 (3.6)

	**Low plasma coenzyme Q10 (*N* = 58)**	**High plasma coenzyme Q10 (*N* = 22)**	***P*-value**

Age (years)	77.2 ± 6.2 (79.0)	74.5 ± 6.2 (76.0)	0.08^†^
Gender (male/female)	16/42	6/16	0.80
**Physical features**			
SBP (mmHg)	129.9 ± 17.4 (132.0)	128.7 ± 18.0 (129.5)	0.79^†^
DBP (mmHg)	73.0 ± 11.0 (71.5)	73.0 ± 8.7 (74.0)	0.98^†^
BMI (kg/m^2^)	24.3 ± 4.0 (23.9)	23.8 ± 3.5 (23.5)	0.58^†^
Waist circumference (cm)	91.5 ± 9.9 (91.0)	88.5 ± 9.9 (88.0)	0.24^†^
Male	92.6 ± 6.2 (92.6)	88.1 ± 8.9 (91.0)	0.20^†^
Female	91.1 ± 11.1 (90.2)	88.7 ± 10.6 (86.0)	0.46^†^
**Clinical characteristics**			
Triglycerides (mmol/L)	1.5 ± 1.1 (1.4)	1.1 ± 0.5 (1.0)	0.06^‡^
Total cholesterol (mmol/L)	4.7 ± 0.9 (4.6)	5.3 ± 1.1 (5.2)	0.02^‡^
LDL-C (mmol/L)	2.9 ± 0.9 (2.7)	3.3 ± 1.0 (3.1)	0.08^‡^
HDL-C (mmol/L)	1.5 ± 0.4 (1.5)	1.8 ± 0.5 (1.8)	<0.01^†^
Male	1.3 ± 0.2 (1.3)	1.7 ± 0.6 (1.7)	0.25^‡^
Female	1.6 ± 0.4 (1.6)	1.9 ± 0.5 (1.9)	0.06^†^
Fasting glucose (mmol/L)	6.5 ± 1.5 (6.0)	6.7 ± 1.9 (6.2)	0.78^‡^
Glycated hemoglobin (%)	6.3 ± 0.8 (6.0)	6.5 ± 1.4 (5.9)	0.54^‡^
Hs-CRP (mg/L)	1.6 ± 1.7 (0.9)	3.5 ± 9.6 (0.8)	0.95^‡^
GOT (U/L)	25.0 ± 11.6 (23.0)	27.5 ± 14.5 (24.5)	0.29^‡^
GPT (U/L)	18.8 ± 8.0 (17.0)	22.0 ± 16.0 (18.0)	0.82^‡^
**Use of hypolipidemic agents (n,%)**			
HMG-CoA reductase inhibitors	22 (37.9%)	8 (36.4%)	0.90
Fibric acid derivatives	4 (6.9%)	0 (0.0%)	0.57
Cholesterol absorption inhibitor	2 (3.4%)	0 (0.0%)	1.00
Nicotinic acid	1 (1.7%)	0 (0.0%)	1.00

Data is shown as mean ± SD (median). Low plasma coenzyme Q10: plasma coenzyme Q10 < 0.5 μM; High plasma coenzyme Q10: plasma coenzyme Q10 ≥ 0.5 μM. ^†^Parametric tests were performed by Student’s *t*-test; ^‡^non-parametric tests were performed by the Mann−Whitney rank sum test. BMI, body mass index; DBP, diastolic blood pressure; GOT, glutamic oxaloacetic transaminase; GPT, glutamic pyruvic transaminase; HDL-C, high density lipoprotein-cholesterol; HMG-CoA, 3-hydroxy-3-methyl- glutaryl-coenzyme A; Hs-CRP, high-sensitivity C-reactive protein; LDL-C, low density lipoprotein-cholesterol; MMSE, mini mental state examination; RBC, red blood cell; SBP, systolic blood pressure; TC, total cholesterol; WBC, white blood cell.

### Antioxidant capacity, biomarkers for dementia, and mini mental state examination score of patients

Table 2 shows the antioxidant capacity, biomarkers for dementia, and MMSE scores of patients after stratification by coenzyme Q10 status. A slightly lower level of serum TAC was found in the patients with low plasma coenzyme Q10 level compared with those with high plasma coenzyme Q10 level (*p* = 0.06), similarly shown in patients with low plasma coenzyme Q10/TC (*p* = 0.08) and low WBC coenzyme Q10 levels (*p* = 0.07). In addition, patients with low WBC coenzyme Q10 level exhibited a significantly lower level of RBC TAC than those with a higher level of WBC coenzyme Q10 (*p* = 0.04). Regarding biomarkers for dementia, patients with low plasma coenzyme Q10/TC (*p* = 0.05) or low WBC coenzyme Q10 levels (*p* = 0.02) exhibited a significantly higher level of serum amyloid β-42. A similar trend was found in the serum amyloid β-42/40 ratio in patients with low plasma coenzyme Q10/TC (*p* = 0.08) or low WBC coenzyme Q10 (*p* = 0.01), while patients with low level of WBC coenzyme Q10 had a slightly lower level of serum amyloid β-40 (*p* = 0.08). However, there was no significant difference in the level of tau protein after stratified by coenzyme Q10 status. In the MMSE score, patients with low coenzyme Q10 status had a significantly lower MMSE score than those with high coenzyme Q10 status (plasma coenzyme Q10, *p* = 0.01; coenzyme Q10/TC, *p* = 0.02).

**TABLE 2:** Antioxidant capacity, biomarkers for dementia, and MMSE score of patients after stratified by coenzyme Q10 status.

	Low plasma coenzyme Q10 (*N* = 58)	High plasma coenzyme Q10 (*N* = 22)	*P*-value
**Antioxidant capacity**			
Serum TAC (mM Trolox)	5.0 ± 0.5 (5.1)	5.2 ± 0.4 (5.3)	0.06^†^
RBC TAC (mM Trolox)	9.5 ± 0.9 (9.6)	9.6 ± 1.1 (9.8)	0.30^‡^
**Biomarkers for dementia**			
Amyloid β-42 (pg/mL)	37.2 ± 12.4 (33.0)	33.8 ± 5.8 (33.0)	0.71^‡^
Amyloid β-40 (pg/mL)	54.5 ± 26.1 (47.4)	46.4 ± 24.3 (46.6)	0.34^‡^
Amyloid β-42/40 ratio	0.92 ± 0.72 (0.78)	1.10 ± 0.99 (0.72)	0.77^‡^
Tau protein (pg/mL)	37.1 ± 28.5 (30.5)	39.3 ± 57.6 (27.6)	0.36^‡^
MMSE score (points)	18.4 ± 5.5 (20.0)	21.5 ± 5.2 (24.0)	0.01^‡^

	**Low plasma coenzyme Q10/TC (*N* = 41)**	**High plasma coenzyme Q10/TC (*N* = 39)**	***P*-value**

**Antioxidant capacity**			
Serum TAC (mM Trolox)	5.0 ± 0.5 (5.1)	5.1 ± 0.4 (5.2)	0.08^†^
RBC TAC (mM Trolox)	9.5 ± 0.8 (9.6)	9.5 ± 1.0 (9.7)	0.54^‡^
**Biomarkers for dementia**			
Amyloid β-42 (pg/mL)	38.6 ± 13.2 (34.0)	33.7 ± 7.3 (32.2)	0.05^‡^
Amyloid β-40 (pg/mL)	50.5 ± 25.2 (45.0)	54.4 ± 26.4 (51.2)	0.37^‡^
Amyloid β-42/40 ratio	1.01 ± 0.78 (0.87)	0.91 ± 0.83 (0.65)	0.08^‡^
Tau protein (pg/mL)	35.4 ± 26.1 (33.0)	40.2 ± 47.6 (27.0)	0.74^‡^
MMSE score (points)	17.9 ± 5.7 (18.0)	20.7 ± 5.1 (23.0)	0.02^‡^

	**Low WBC coenzyme Q10 (*N* = 39)**	**High WBC coenzyme Q10 (*N* = 41)**	***P*-value**

**Antioxidant capacity**			
Serum TAC (mM Trolox)	5.0 ± 0.5 (5.0)	5.1 ± 0.4 (5.3)	0.07^†^
RBC TAC (mM Trolox)	9.3 ± 0.9 (9.4)	9.7 ± 0.9 (9.7)	0.04^‡^
**Biomarkers for dementia**			
Amyloid β-42 (pg/mL)	39.5 ± 13.6 (34.5)	33.1 ± 6.4 (31.9)	0.02^‡^
Amyloid β-40 (pg/mL)	47.3 ± 24.3 (45.5)	57.3 ± 26.4 (52.2)	0.08^‡^
Amyloid β-42/40 ratio	1.13 ± 0.85 (0.86)	0.80 ± 0.71 (0.64)	0.01^‡^
Tau protein (pg/mL)	34.5 ± 24.3 (30.6)	40.8 ± 47.6 (27.0)	0.76^‡^
MMSE score (points)	19.7 ± 5.1 (21.0)	18.9 ± 6.0 (20.0)	0.63^‡^

Data is shown as mean ± SD (median). Low plasma coenzyme Q10: plasma coenzyme Q10 < 0.5 μM; High plasma coenzyme Q10: plasma coenzyme Q10 ≥0.5 μM. Low plasma coenzyme Q10/TC: plasma coenzyme Q10/TC < median value; High plasma coenzyme Q10/TC: plasma coenzyme Q10/TC ≥ median value. Low WBC coenzyme Q10: WBC coenzyme Q10< median value; High WBC coenzyme Q10: WBC coenzyme Q10≥ median value. ^†^Parametric tests were performed by Student’s *t*-test; ^‡^non-parametric tests were performed by the Mann−Whitney rank sum test. MMSE, mini mental state examination; RBC, red blood cell; TAC, total antioxidant capacity; TC, total cholesterol; WBC, white blood cell.

### Correlations between biomarkers for dementia and antioxidant capacity

**FIGURE 1:**
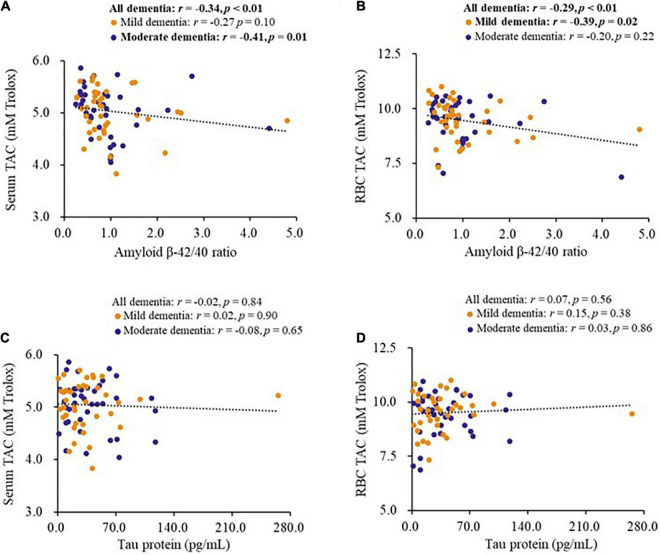
Correlations between biomarkers for dementia and antioxidant capacity of patients. **(A,B)** Correlations between the level of amyloid β-42/40 ratio and antioxidant capacity. **(C,D)** Correlations between the level of tau protein and antioxidant capacity. CoQ10, Aβ, Tau in Dementia.

The correlations between biomarkers for dementia and antioxidant capacity are shown in Figure 1. The level of serum amyloid β-42 (*r* = −0.25, *p* = 0.02), serum amyloid β-40 (*r* = 0.29, *p* = 0.01), and amyloid β-42/40 ratio (*r* = −0.34, *p* < 0.01) were significantly correlated with the level of serum TAC, as well as in the patients with moderate dementia (Figure 1A). In addition, the level of serum amyloid β-40 (*r* = 0.33, *p* < 0.01) and amyloid β-42/40 ratio (*r* = −0.29, *p* < 0.01, Figure 1B) were significantly correlated with the level of RBC TAC, and the significant correlations were also found in patients with mild dementia (Figure 1B). However, there was no significant correlation between tau protein and antioxidant capacity (Figures 1C,D).

### Correlations between coenzyme Q10 status and antioxidant capacity

**FIGURE 2:**
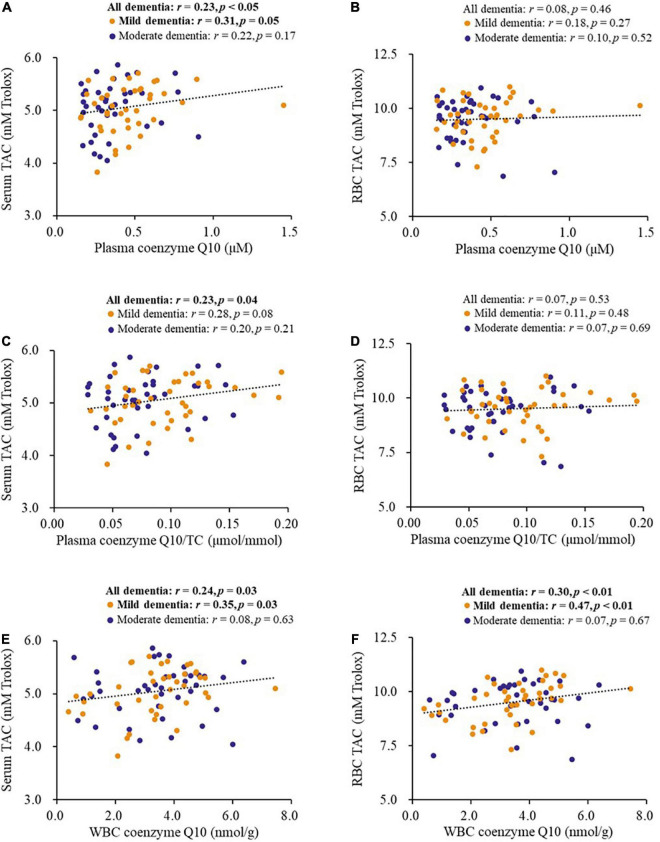
Correlations between coenzyme Q10 status and antioxidant capacity of patients. **(A,B)** Correlations between the level of plasma coenzyme Q10 and antioxidant capacity. **(C,D)** Correlations between the level of plasma coenzyme Q10/TC and antioxidant capacity. **(E,F)** Correlations between the level of WBC coenzyme Q10 and antioxidant capacity. CoQ10, Aβ, Tau in Dementia.

Figure 2 shows the correlations between coenzyme Q10 status and antioxidant capacity in patients with dementia. Coenzyme Q10 status was significantly positively correlated with the levels of serum TAC (plasma coenzyme Q10, *r* = 0.23, *p* < 0.05, Figure 2A; plasma coenzyme Q10/TC, *r* = 0.23, *p* = 0.04, Figure 2C; WBC coenzyme Q10, *r* = 0.24, *p* = 0.03, Figure 2E) and RBC TAC (WBC coenzyme Q10, *r* = 0.30, *p* < 0.01, Figure 2F). These significant correlations also existed in patients with mild dementia (Figures 2A,E,F).

### Correlations between coenzyme Q10 status and biomarkers for dementia

**FIGURE 3:**
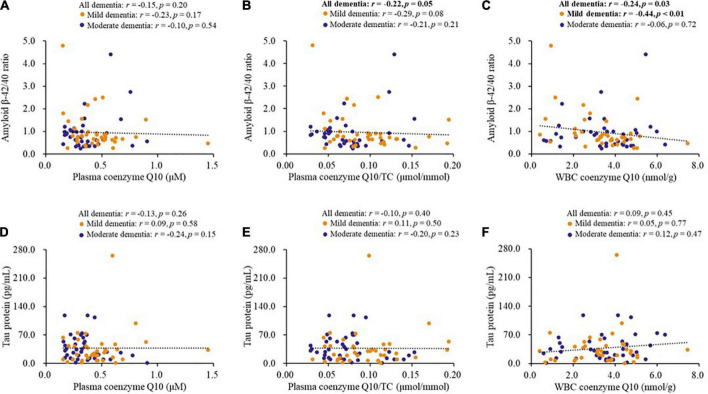
Correlations between coenzyme Q10 status and biomarkers for dementia of patients. **(A–C)** Correlations between coenzyme Q10 status and the level of amyloid β-42/40 ratio. **(D–F)** Correlations between coenzyme Q10 status and the level of tau protein. CoQ10, Aβ, Tau in Dementia.

Figure 3 shows the correlations between coenzyme Q10 status and biomarkers for dementia. Coenzyme Q10 status was significantly correlated with the levels of serum amyloid β-42 (plasma coenzyme Q10/TC, *r* = −0.28, *p* = 0.01; WBC coenzyme Q10, *r* = −0.26, *p* = 0.02) and the amyloid β-42/40 ratio (plasma coenzyme Q10/TC, *r* = −0.22, *p* = 0.05, Figure 3B; WBC coenzyme Q10, *r* = −0.24, *p* = 0.03, Figure 3C), and these correlations also existed in patients with mild dementia for the amyloid β-42/40 ratio (*r* = −0.44, *p* < 0.01, Figure 3C) or with moderate dementia for the amyloid β-42 (*r* = −0.37, *p* = 0.02). However, there was no significant correlation between coenzyme Q10 status and tau protein (Figures 3D–F).

### Correlations between coenzyme Q10 status and mini mental state examination score

**FIGURE 4:**
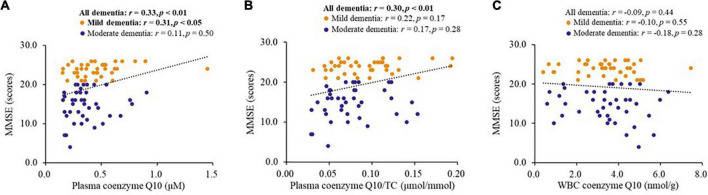
Correlations between coenzyme Q10 status and the MMSE score of patients. CoQ10, Aβ, Tau in Dementia. **(A)** Correlation between the level of plasma coenzyme Q10 and MMSE score. **(B)** Correlation between the level of plasma coenzyme Q10/TC and MMSE score. **(C)** Correlation between the level of WBC coenzyme Q10 and MMSE score.

Figure 4 shows the correlations between coenzyme Q10 status and MMSE score in patients with dementia. Coenzyme Q10 status was significantly correlated with MMSE score (plasma coenzyme Q10, *r* = 0.33, *p* < 0.01, Figure 4A; plasma coenzyme Q10/TC, *r* = 0.30, *p* < 0.01, Figure 4B). Plasma coenzyme Q10 level was significantly correlated with MMSE score in patients with mild dementia (*r* = 0.31, *p* < 0.05, Figure 4A).

### Coenzyme Q10 status and dementia progression

**FIGURE 5:**
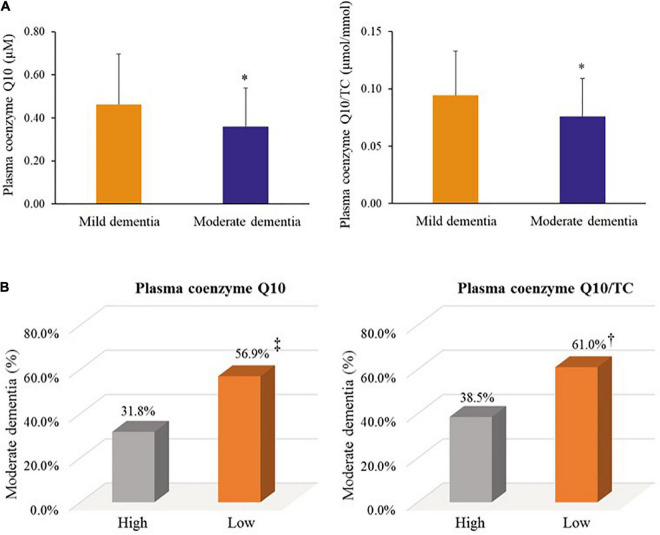
Coenzyme Q10 status and dementia progression. **(A)** Coenzyme Q10 status in patients with mild and moderate dementia. **(B)** The proportion of moderate dementia by coenzyme Q10 status. Mild dementia: the score of MMSE > 20; Moderate dementia: the score of MMSE ≤ 20. Low plasma coenzyme Q10: plasma coenzyme Q10 < 0.5 μM; High plasma coenzyme Q10: plasma coenzyme Q10 ≥ 0.5 μM. Low plasma coenzyme Q10/TC: plasma coenzyme Q10/TC < median value; High plasma coenzyme Q10/TC: plasma coenzyme Q10/TC ≥ median value. MMSE, mini mental state examination; TC, total cholesterol; WBC, white blood cell. *, *p* < 0.05; ^†^, *p* = 0.07; ^‡^, *p* = 0.08.

We further examined coenzyme Q10 status and dementia progression, as shown in Figure 5. Patients with moderate dementia had a significantly lower level of coenzyme Q10 status than those with mild dementia (plasma coenzyme Q10, median value: 0.32 vs. 0.45 μM, *p* = 0.01; plasma coenzyme Q10/TC, median value: 0.07 vs. 0.09 μmol/mmol, *p* = 0.03, Figure 5A). In addition, patients with low level of plasma coenzyme Q10 (56.9 vs. 31.8%, *p* = 0.08) or low level of plasma coenzyme Q10/TC (61.0 vs. 38.5%, *p* = 0.07) showed a slightly higher proportion of moderate dementia than those with high coenzyme Q10 status (Figure 5B).

## Discussion

This study was the first to investigate the relationship between coenzyme Q10 status and biomarkers in patients with dementia. The amyloid hypothesis of the pathogenesis of dementia was proposed by John Hardy and David Allsop in 1991 (Hardy and Allsop, 1991). Amyloid β is produced through the amyloidogenic pathway (Coronel et al., 2018), and amyloid β-42 and amyloid β-40 are produced by cleavage of Aβ precursor protein by γ-secretase (Coronel et al., 2018; Fan et al., 2020). In this study, we found that patients with higher coenzyme Q10 status had a significantly lower level of serum amyloid β-42 and amyloid β-42/40 ratio (Table 2), and they were significantly correlated with each other (Figure 3). Amyloid β is proposed to induce hyperphosphorylation of tau protein during the pathological severity of dementia (Mocanu et al., 2008; Fan et al., 2020). Studies in the elderly population have found that amyloid β is related to neuropathological performance (Yun et al., 2021; Zecca et al., 2021). The neuroprotective effects of coenzyme Q10 have also been demonstrated in cells and animal models (Elipenahli et al., 2012; Komaki et al., 2019; Ekicier Acar et al., 2020; Ibrahim Fouad, 2020). Administration of coenzyme Q10 could attenuate amyloid β accumulation and mitigate Alzheimer’s-like behavioral and pathological symptoms (Yang et al., 2008; Muthukumaran et al., 2018; Ibrahim Fouad, 2020). Recent published data showed that lower levels of amyloid β-42 and amyloid β-42/40 ratio was found in dementia patients (Hanon et al., 2022; Thijssen et al., 2022), but other researchers found that patients with dementia had higher levels of amyloid β-42, and the level of plasma amyloid β-42/40 ratio was correlated with amyloid positivity in the bilateral frontal, parietal, temporal, and occipital cortices (Manafikhi et al., 2021; Yun et al., 2021). The levels of amyloid β-42 and β-40 in dementia patients appear to remain uncertain. Administration of coenzyme Q10 reduced the level of serum amyloid β-42 and showed neuroprotective effects in animal experiments (Ibrahim Fouad, 2020). Further human studies are needed to elucidate the effect of CoQ10 supplementation on biomarkers of dementia. Although we did not find a significant correlation between coenzyme Q10 status and tau protein in the present study, Yang et al. (2020) demonstrated that coenzyme Q10 could reduce the expression of phosphorylated tau protein and attenuate neuroinflammation. As plasma total tau protein just partly reflects the pathology of dementia (Mattsson et al., 2016), evidence has indicated that measurements of phosphorylated tau species, P-tau181, P-tau217, and P-tau231, appear to have better performance in discriminating patients with cognitive impairment (Tang et al., 2018; Palmqvist et al., 2021; Tissot et al., 2022; Verde, 2022). Therefore, future studies should attempt to examine the correlation between coenzyme Q10 and phosphorylated tau instead of total tau protein. Additionally, it is worth noting that 73% of patients in this study suffered from coenzyme Q10 deficiency (plasma coenzyme Q10 < 0.5 μM, Molyneux et al., 2008). In addition to the disease, aging is also a risk factor for impaired coenzyme Q10 status (Díaz-Casado et al., 2019). Because it is not easy to include the age matching healthy controls, so we tried to compare the level of coenzyme Q10 with our previous study that the middle-aged and elderly without osteoarthritis (Chang et al., 2020), and we found that coenzyme Q10 status was lower in the aging and patients with dementia (Median level of plasma coenzyme Q10, Dementia vs. Middle and Elderly, 0.36 vs. 0.45 μM, *p* < 0.01). However, the middle-aged and elderly without osteoarthritis in our previous study without assessing MMSE scores, so we did not make comparisons in the present study. Therefore, it is necessary to monitor coenzyme Q10 status in patients with dementia, and dietary supplementation with coenzyme Q10 could be considered to increase coenzyme Q10 to normal levels.

The oxidative stress hypothesis of dementia pathogenesis has been widely discussed (Butterfield and Halliwell, 2019; Fracassi et al., 2021). Growing evidence supports that oxidative stress, such as DNA damage, Aβ accumulation, tau hyperphosphorylation, subsequent mitochondrial dysfunction, and loss of synapses and neurons, is an essential part of the development of dementia (Chen and Zhong, 2014; Wang et al., 2014; Butterfield and Halliwell, 2019). Antioxidants may be beneficial in complementary therapy for dementia (Chen and Zhong, 2014). Coenzyme Q10 is considered to be a good antioxidant in mitochondria and membranes, which provides its neuroprotective effects by inhibiting oxidative stress (Choi et al., 2012). In human neuronal cells, coenzyme Q10 level is related to neuronal mitochondrial function and oxidative stress, and mitochondrial oxidative stress can be attenuated after coenzyme Q10 supplementation (Duberley et al., 2013, 2014). In this study, we used the WBC sample, which has a nucleus, as an estimate of coenzyme Q10 in tissue (Duncan et al., 2005). In the present study, we found that amyloid β-42 and the amyloid β-42/40 ratio were negatively correlated with TAC (Figure 1), while coenzyme Q10 status was positively correlated with the level of TAC (Table 2 and Figure 2). These results may imply that an improvement in coenzyme Q10 status is related to a good antioxidant capacity in patients with dementia. Notably, most of the significant correlations were also found in patients with mild dementia (Figures 1–3). Coenzyme Q10 may exert neuroprotective effects by its antioxidant capacity against the accumulation of amyloid-β on synaptic plasticity in the hippocampus (Yang et al., 2008; Komaki et al., 2019; Ibrahim Fouad, 2020). Recently, the pathologic development of dementia was associated with free-radical production causing the formation of β-amyloid aggregates, research demonstrated some lipid peroxidation metabolites could be potential biomarkers in patients with dementia (Peña-Bautista et al., 2019). In order to reduce the invasive and expensive diagnosis techniques for the diagnosis of dementia, a series of plasma lipid peroxidation biomarkers that could reflect brain damage have been validated as a satisfactory early diagnostic model (Zuliani et al., 2018). In addition, high level of lipid peroxidation was usually accompanied with defecting the antioxidants status (Manoharan et al., 2016). Coenzyme Q10 acting as a lipophilic antioxidant could exert its antioxidant capacity in patients (Figure 2) and is beneficial for the progression of dementia (Figures 4, 5). As a result, examining the effects of coenzyme Q10 supplement on these redox biomarkers in patients with dementia can be the next step for the research.

In the present study, we also found that coenzyme Q10 status was correlated with cognitive performance MMSE in patients with dementia (Figure 4). Not only did patients with moderate dementia show a significantly lower coenzyme Q10 status, but a higher proportion of patients with low coenzyme Q10 status suffered from moderate dementia (Figure 5). The results are also supported by the Japanese general population (Community Circulation Risk Study), which reported that low level of coenzyme Q10 was associated with dementia risk and suggested that coenzyme Q10 level may be a predictor for the development of dementia, rather than a biomarker for the presence of dementia (Momiyama, 2014; Yamagishi et al., 2014). Recently, two clinical trials have been conducted to investigate the effect of coenzyme Q10 supplementation on cognitive evaluation (Ramezani et al., 2020; García-Carpintero et al., 2021). Patients with acute ischemic stroke treated with coenzyme Q10 supplementation (300 mg/day) for 4 weeks showed an improvement in MMSE score (Ramezani et al., 2020). Another clinical study found that ubiquinol supplementation (200 mg/day) improved cerebral vasoreactivity and ameliorated chronic inflammation in patients with mild cognitive impairment (García-Carpintero et al., 2021). In addition, it is interesting to note that a total of 36.3% of dementia patients suffered from diabetes, 26.3% were suffered from prediabetes in the present study. Moreover, the levels of glucose parameters were correlated with systolic blood pressure (fasting glucose: *r* = 0.32, *p* < 0.01; glycated hemoglobin: *r* = 0.28, *p* = 0.01) and triglycerides (fasting glucose: *r* = 0.33, *p* < 0.01; glycated hemoglobin: *r* = 0.41, *p* < 0.01) in patients with dementia. Glycemic disorders have been identified as a key risk factor for dementia (Pal et al., 2018). Recent clinical studies demonstrated that coenzyme Q10 supplementation is beneficial for glycemic control, especially in diabetes (Yen et al., 2018; Yoo and Yum, 2018; Zhang et al., 2018; Gholami et al., 2019). A dose of 100–200 mg/d of coenzyme Q10 supplement for 8–12 weeks seems could significantly improve the insulin resistance and the level of glucose parameters, in patients with prediabetes, type 2 diabetes (Yen et al., 2018; Yoo and Yum, 2018; Gholami et al., 2019), or in dyslipidemia individuals (Zhang et al., 2018). Since coenzyme Q10 status may be associated with delayed cognitive decline and improved glycemic control, it is worth further interventional studies to verify the dosage and formulation of coenzyme Q10 supplementation and examine the effect on cognitive performance and glycemic control in dementia patients.

The limitations of this study include that we cannot confirm the types of dementia, such as Alzheimer’s disease or vascular dementia. Second, this is a single-center, cross-sectional study, and only a correlation but not a causal relationship between coenzyme Q10 and biomarkers for dementia can be established from the results. Third, we did not measure the cerebral level of coenzyme Q10 and amyloid-β in the present study because it is not easy to access in clinical sampling. However, some animal models evidence found that treatment with coenzyme Q10 could provide beneficial effects on the brain (Yang et al., 2008; Muthukumaran et al., 2018; Ibrahim Fouad, 2020). Thus, clinical interventional studies are needed to validate the results in future studies.

## Conclusion

**FIGURE 6:**
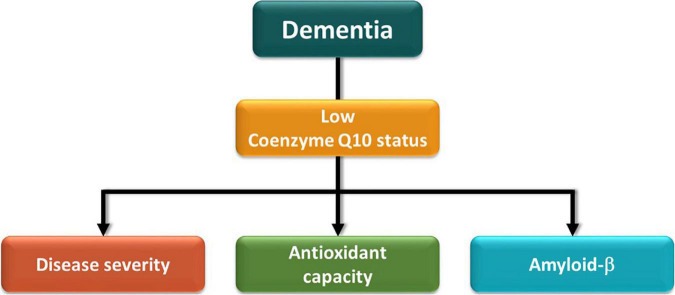
Schematic representation of concluding remark.

The concluding remark of the present study is schematically summarized in Figure 6. This study found that patients with dementia suffered from a low coenzyme Q10 status, and the level of coenzyme Q10 was significantly correlated with the level of amyloid β-42 and the amyloid β-42/40 ratio, but not with the level of tau protein. In addition, both coenzyme Q10 status and amyloid-β levels were related to antioxidant capacity in patients with dementia. Since the level of coenzyme Q10 may be associated with delayed the progression of dementia, monitoring the level of coenzyme Q10 in patients with dementia is necessary. Further intervention studies will elucidate the causal effects of coenzyme Q10 supplementation on these parameters for dementia.

## Data availability statement

The raw data supporting the conclusions of this article will be made available by the authors, without undue reservation.

## Ethics statement

The studies involving human participants were reviewed and approved by the Institutional Review Board of Chung Shan Medical University Hospital, Taiwan (CSMUH No: CS2-18147). The patients/participants provided their written informed consent to participate in this study.

## Author contributions

P-SC, H-HC, T-JL, and C-HY performed the study and recruited the subjects. P-SC performed the data analyses. J-CP helped perform the study and analyzed the sample. P-TL conceived the study, participated the design, and coordinated the study. P-SC and P-TL drafted the manuscript. All authors read and approved the final manuscript.

## Abbreviations

BMI: body mass index
HDL-C: high density lipoprotein-cholesterol
HPLC: high performance liquid chromatography
MMSE: mini mental state examination
RBC: red blood cell
TAC: total antioxidant capacity
TC: total cholesterol
TG: triglycerides
WBC: white blood cell.
